# First person – Tuba Sural-Fehr

**DOI:** 10.1242/dmm.040402

**Published:** 2019-05-23

## Abstract

First Person is a series of interviews with the first authors of a selection of papers published in Disease Models & Mechanisms (DMM), helping early-career researchers promote themselves alongside their papers. Tuba Sural-Fehr is first author on ‘Inhibition of the IGF-1–PI3K–Akt–mTORC2 pathway in lipid rafts increases neuronal vulnerability in a genetic lysosomal glycosphingolipidosis’, published in DMM. Tuba conducted the research described in this article while a Postdoctoral Fellow in Dr Ernesto Bongarzone's lab at University of Illinois-Chicago, Chicago, USA.


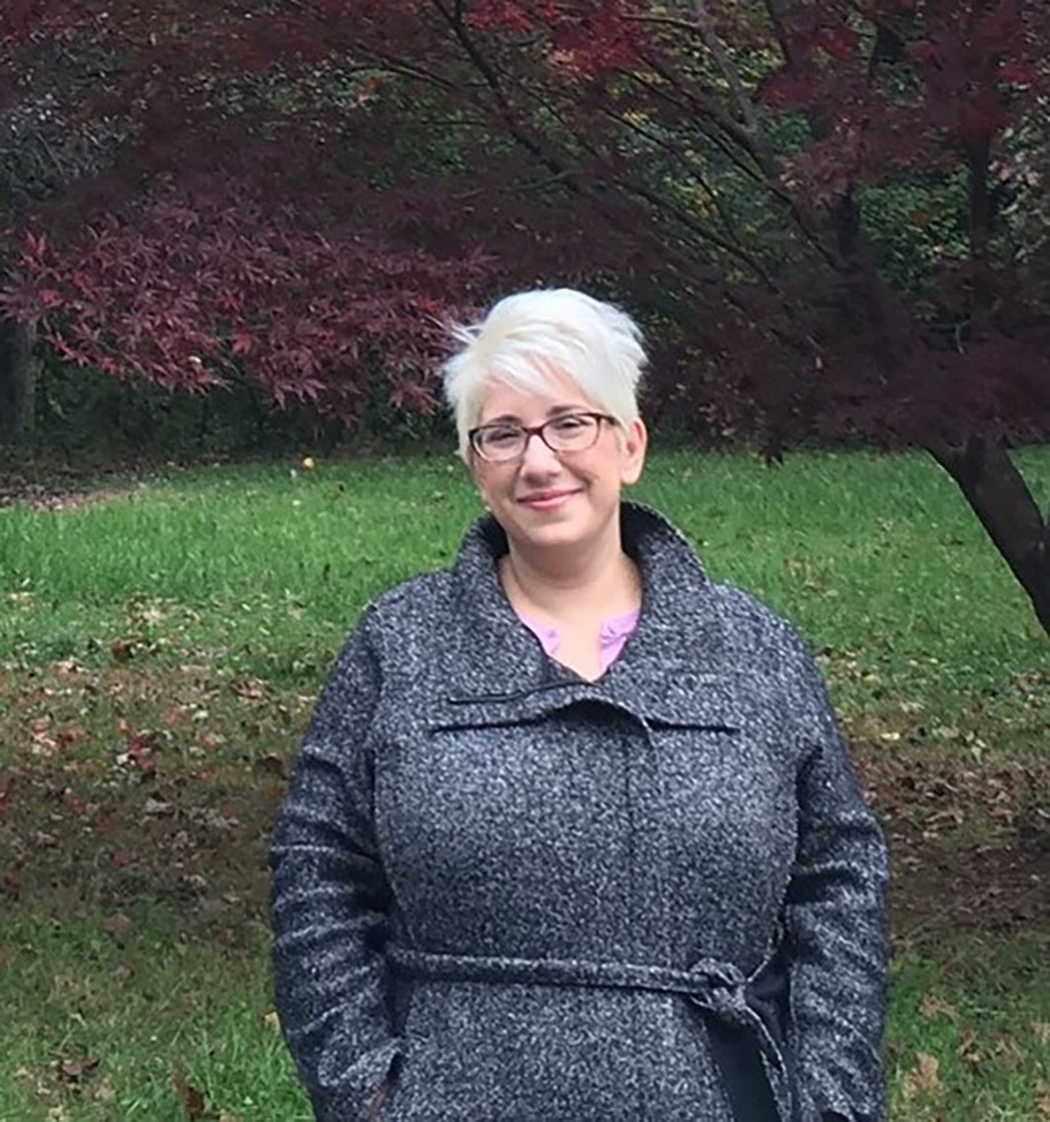


**Tuba Sural-Fehr**

**How would you explain the main findings of your paper to non-scientific family and friends?**

Krabbe disease (KD) is a rare, inherited neurological disorder that results in progressive damage to the nervous system due to the accumulation of a toxic chemical called psychosine. There is no cure for this disease, and bone marrow transplantation (BMT) or umbilical cord blood transplantation are the only treatments that extends a child's life, although these have been limited in preventing subsequent neurological deficits. This suggests that we need additional therapies to treat the underlying defects that are not corrected with BMT. In this study, we sought to understand how psychosine accumulation in KD becomes toxic to neurons in the hope of identifying new treatments that could improve neurological outcomes for these patients. For this, we used the twitcher KD mouse model and cultured cells to look at the effects of psychosine accumulation on the health and survival of neurons. We found that psychosine, slowly and over time, inhibits a neuron's ability to interpret external signals that instruct it to grow and survive. It does so specifically at the cell membrane by blocking incoming survival signals from reaching messengers that deliver it to the cell's interior. We also identified a time window after which psychosine's harmful effects become irreversible and were able to show that supplementing cells with a key insulin-like molecule can improve survival if given within this time window.

**What are the potential implications of these results for your field of research?**

Our work predicts that signaling pathways that rely mainly on membrane lipid raft integrity in any given cellular context would be affected by psychosine treatment, especially growth-factor-mediated receptor tyrosine kinases (RTKs) and chemokine-mediated G protein-coupled receptors (GPCRs). We hypothesize that, depending on which pathways are active in any given cell type at any given time, the ones that depend significantly on lipid rafts for activation and/or continued activity will be sensitive to psychosine exposure. Interestingly, the fact that psychosine exposure does not completely shut down Akt phosphorylation, and that IGF-1R pathway components are intact and inhibition is reversible under certain conditions, offers a window of opportunity for therapeutic interventions. An attractive idea for therapy would be to treat patients with IGF-1 and/or other growth factors to improve intracellular signaling and therefore cellular resilience to psychosine. Our data suggests that this treatment should be done in conjunction with hematopoietic stem cell transplantation or gene therapy, initiated at the earliest time point possible, and continued for a specified period of time after genetic correction. Since psychosine inhibition originates at the cell membrane and likely has a domino effect on multiple downstream cellular processes, future therapies may benefit from drug screens directed at more upstream targets at the membrane; even preserving the structure of the cell membrane itself could be a therapeutic approach.

“Some institutions do a good job of exposing their trainees to a variety of options through career and professional development events, but getting hands-on real-world experience is hard.”

**What has surprised you the most while conducting your research?**

It was surprising to see that inhibition of signaling pathways by psychosine starts during embryogenesis much earlier than previously thought, which may explain why some Krabbe babies are born already showing symptoms. This has implications for the timing of future therapies, which may need to be initiated *in utero* instead of after birth.

**Figure:**
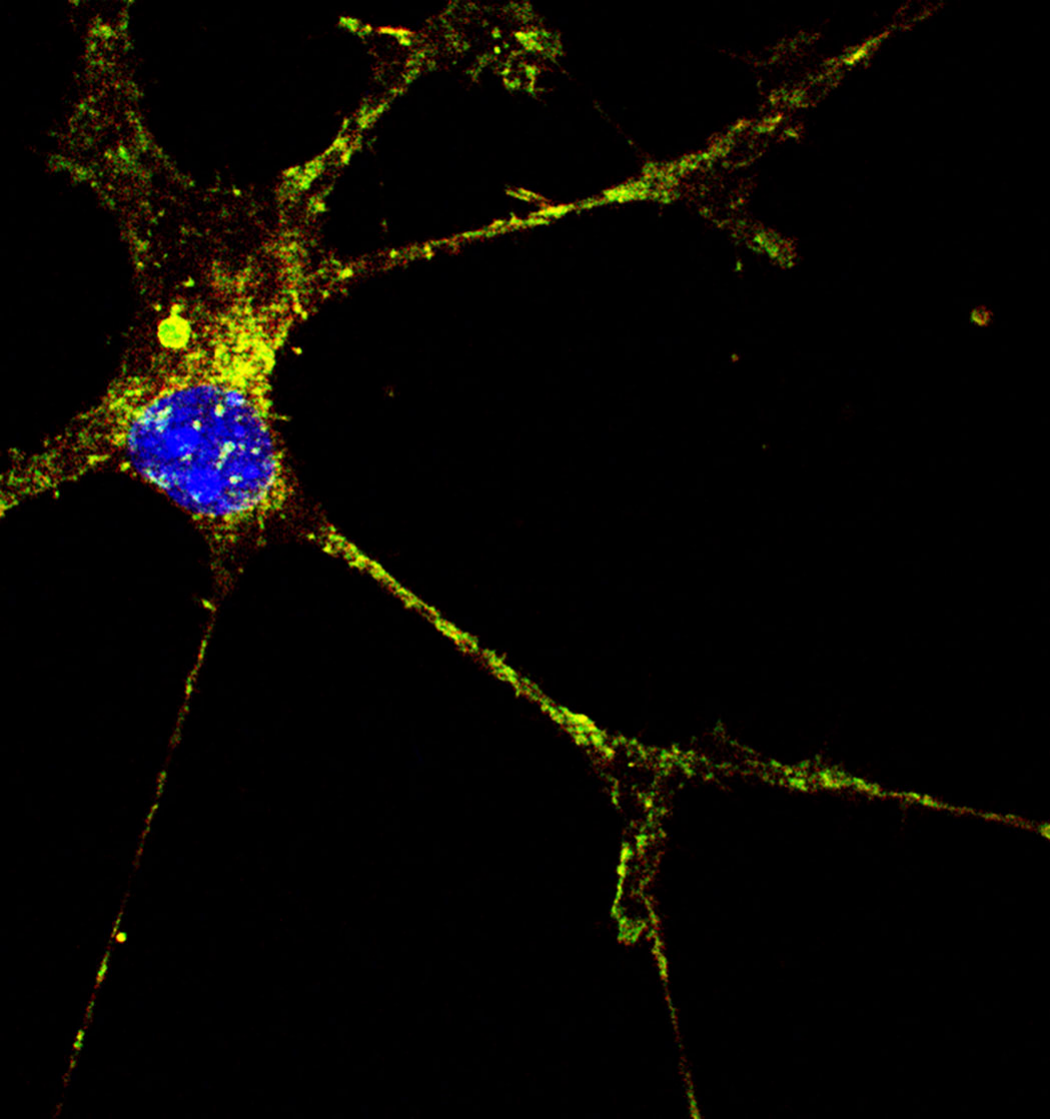
**A twitcher neuronal cell stained for IGF-1R- and cholera-toxin-positive lipid rafts, demonstrating that, in disease, localization of receptors to lipid rafts is not affected.**

**What changes do you think could improve the professional lives of early-career scientists?**

With limited tenure-track positions available these days, most graduate students and postdocs will end up transitioning away from the bench, but they are not encouraged to explore those options for fear of becoming a ‘failure’ of the academic system. Therefore, opportunities to explore career options beyond the bench are often missing in our training system, especially for postdocs. Some institutions do a good job of exposing their trainees to a variety of options through career and professional development events, but getting hands-on real-world experience is hard. Having opportunities for mini-internships outside of the lab would benefit early-career scientists by not only exposing them to different career paths, but also by allowing them to gain transferrable skills to make that transition easier.

**What's next for you?**

During my postdoc work on KD, I met families and their children affected by this disease and became interested in policy and public health aspects of the research that I was doing. I started asking bigger questions, such as: “Would you screen newborns for a genetic condition, such as KD, knowing there were no highly effective treatments?”, “Why is KD being screened at birth in some states and not in others?”, “Should we be screening babies in the prenatal period instead?”, “Would it change the argument to advocate for universal screening if a new treatment was on the horizon?”. After completing my postdoc, I moved to the National Institutes of Health (NIH) as an AAAS Science & Technology Policy Fellow to learn the answers to those questions as it relates to federal policymaking and how federal policies affect the everyday conduct of research and inform public health. In my current office as a science policy fellow, I help oversee Newborn Screening-related programs and initiatives at the NIH and, in collaboration with our partner federal agencies, learn about how scientific evidence is generated and utilized to inform decision making for public health programs such as Newborn Screening.
